# Knitted 3D Scaffolds of Polybutylene Succinate Support Human Mesenchymal Stem Cell Growth and Osteogenesis

**DOI:** 10.1155/2018/5928935

**Published:** 2018-05-07

**Authors:** Miina Ojansivu, Laura Johansson, Sari Vanhatupa, Ilmari Tamminen, Markus Hannula, Jari Hyttinen, Minna Kellomäki, Susanna Miettinen

**Affiliations:** ^1^Adult Stem Cell Research Group, Faculty of Medicine and Life Sciences and BioMediTech Institute, University of Tampere, Tampere, Finland; ^2^Science Center, Tampere University Hospital, Tampere, Finland; ^3^Laboratory of Biomaterials and Tissue Engineering, Faculty of Biomedical Science and Engineering and BioMediTech Institute, Tampere University of Technology, Tampere, Finland; ^4^The Computational Biophysics and Imaging Group, Faculty of Biomedical Science and Engineering and BioMediTech Institute, Tampere University of Technology, Tampere, Finland

## Abstract

Polybutylene succinate (PBS) is a biodegradable polyester with better processability and different mechanical properties compared to polylactides (PLAs), the most commonly used synthetic polymers in tissue engineering (TE). Since only few studies have evaluated PBS-containing materials for bone TE, we prepared PLA-PBS blends and analyzed material properties as well as cell attachment, proliferation, and osteogenic differentiation of human mesenchymal stem cells (hMSCs) on scaffolds. In addition to PLA, PBS, and PLA-PBS blends, PLA-polycaprolactone and PLA-poly(trimethylene carbonate) blends were evaluated. Polymer fibers were prepared using melt spinning. Pure PBS was observed to have the highest crystallinity and strain at break compared to the tougher PLA and PLA blends. No degradation occurred during the 4-week hydrolysis in either of the materials. Knitted and rolled scaffolds were manufactured, seeded with hMSCs, and cultured for 27 days. Human MSC viability was good on all the materials, but cell spreading along the fibers was only detected in PBS-containing scaffolds. They also induced the strongest proliferative response and osteogenic differentiation, which diminished with decreasing PBS content. Based on these results, PBS is superior to PLA with respect to hMSC attachment, proliferation, and osteogenesis. This encourages utilizing PBS-based biomaterials more widely in bone TE applications.

## 1. Introduction

In search for an optimal biomaterial for bone tissue engineering (TE) applications, an increasing number of varying biomaterial formulations and structures have been evaluated during the past decades. In order to be suitable for bone regeneration, the material should fulfill a list of requirements which includes biocompatibility, biodegradability, sufficient mechanical strength, and ability to promote cell adhesion, proliferation, and osteogenic differentiation [1, 2]. Although not a single biomaterial is likely to fulfill all the criteria, certain polymers have been observed to perform with a satisfactory fashion in the bone regeneration applications. Among these are several synthetic polymers, such as aliphatic poly-*α*-hydroxy esters polylactic acid (PLA), polyglycolic acid (PGA), and their copolymer, poly(lactic-co-glycolic acid) (PLGA) [2, 3]. However, despite their established position in bone fixation and promising results in the field of bone TE, these materials possess also drawbacks, such as problems related to hydrophobicity, processability, and release of acidic degradation products. This has increased the interest in alternative polymers for bone regeneration applications.

Polybutylene succinate (PBS), a biodegradable aliphatic polyester, has excellent mechanical and thermal properties, good processability, and low cost, which have made it an attractive material for various purposes [4]. From the beginning of the 1990s, PBS has been commercially produced for biodegradable package material [4], but during recent years, a question has been raised about its suitability for biomedical applications as well. Indeed, with respect to cell attachment, viability, and proliferation, pure PBS as well as PBS-chitosan and PBS-*β*-tricalcium phosphate composites have shown promising results with mouse and rat fibroblastic and osteoblastic cells as well as with human mesenchymal stem cells (hMSCs) [5–10]. However, only few reports have evaluated the cellular response of osteogenic differentiation on PBS-containing materials. Specifically, Li et al. reported a comparable alkaline phosphatase (ALP) activity in rat osteoblasts on PBS discs and on polystyrene control [8]. Wang et al., on the other hand, observed increased osteogenesis of rat osteoblasts on PBS surfaces modified by plasma immersion ion implantation when compared to nontreated surfaces, but no comparison was made between PBS and other polymers [11]. Moreover, 3D porous compression-moulded PBS-chitosan composites were reported to support bone formation both *in vitro* and *in vivo* in a mouse critical-sized calvarial defect model [12, 13]. This data suggests that PBS might have potential for bone TE applications.

In order to tailor the properties of polymers to meet the desired criteria, blending of different polymers is often conducted [14]. In most cases, blending of two different polymers results in material properties with an average of the original polymers. This gives very interesting possibilities to easily customize a material for certain applications. For example, blending of PBS with chitosan has resulted in a favorable outcome with respect to cell attachment, viability, proliferation, and ALP activity [6, 12]. There is also evidence that, upon subcutaneous implantation in rats, the fibrous capsule thickness is smaller with discs of PLA-PBS (50/50 wt%) blend than with either pure PLA or pure PBS [15]. Therefore, blending of polymers is an attractive choice when developing novel functional biomaterials for bone TE.

When considering the 3D architecture of the TE scaffolds, textile-based manufacturing strategies produce inherently porous and interconnected structures with high reproducibility and the possibility to easily scale up the production [10]. However, despite these advantages, textile technology is still a relatively new approach in the field of TE and it offers plenty of unexplored possibilities. For example, with respect to PBS, weft-knitted 2D constructs have been shown to support the attachment of mouse fibroblastic L929 cells [5], but the feasibility of PBS in textile-based structures for supporting osteogenic differentiation has not been evaluated.

In this study, human adipose stem cells (hASCs), multipotent hMSCs easily isolated from adipose tissue, were cultured in knitted and rolled 3D scaffolds prepared from PBS and PLA-PBS blends of 5 and 25 wt% PBS. Pure PLA as well as PLA blends of 5 wt% polycaprolactone (PCL) or poly(trimethylene carbonate) (PTMC) were used as reference materials. PCL is a FDA-approved material widely used in the applications of regenerative medicine and bone TE [16]. Also PTMC, a biocompatible and biodegradable polymer with nonacidic degradation products, has recently gained attention in bone regeneration, especially in the form of membranes and as a drug carrier [17, 18]. The 5 wt% blend composition was chosen because with this composition it was possible to obtain the same mixture ratio in all the material combinations and still be able to produce the fiber. PLA scaffolds of the same architecture are commercially available as joint implants (RegJoint™, Scaffdex, Tampere, Finland), and as PLA-chitosan and PLA-bioactive glass composites, these scaffolds have been also evaluated for chondrogenic differentiation of rabbit ASCs [19]. However, the knitted and rolled scaffolds have not been previously manufactured from other polymers or assessed for the purpose of bone TE. After characterizing the material properties (degradation and thermal and mechanical properties), the viability, attachment, and proliferation of hASCs in the scaffolds were evaluated. Moreover, the osteogenic differentiation of hASCs within the scaffolds was assessed by determining the ALP activity, osteogenic marker gene expression (*RUNX2a*, *OSTERIX*, and *DLX5*), and formation of CaP mineral. To our knowledge, osteogenic differentiation has not been previously analyzed in 3D structures of pure PBS or PLA-PBS blends.

## 2. Materials and Methods

### 2.1. Ethics Statement

This study was conducted in accordance with the Ethics Committee of the Pirkanmaa Hospital District, Tampere, Finland (R15161). The hASCs were isolated from adipose tissue samples obtained from surgical procedures conducted in the Department of Plastic Surgery, Tampere University Hospital. There were five women donors of age 50 ± 17 years. All the donors gave a written informed consent for the utilization of the adipose tissue samples in research settings.

### 2.2. Scaffold Manufacturing

**Figure 1:**
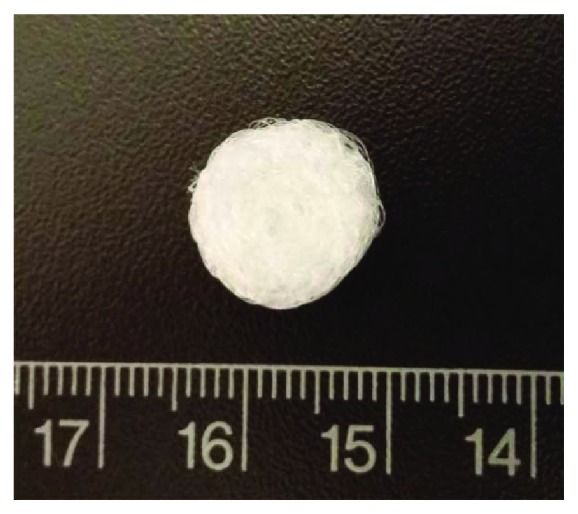
A representative image of a knitted 3D scaffold.

The materials used in this study were poly(L/D)lactide 96/4 (PLA) copolymer (Purasorb PLD 9620, Purac Biochem BV, Gorinchem, Netherlands), poly-*ε*-caprolactone (PCL) polymer (Purasorb PC 12, Purac Biochem BV), polybutylene succinate (PBS) copolymer (Bionolle 1020 MD, Showa Denko Europe GmbH, Munich, Germany), and poly (trimetylene carbonate) (PTMC) polymer. PTMC was kindly provided by Professor Dirk Grijpma from the University of Twente. The inherent viscosities of the raw polymers used are presented in Table 1. The polymers were blended and fiber spun in a two-stage process. Before both stages, the polymers were dried in vacuum. First, the blending was done using a custom-made laboratory scale corotating twin-screw extruder in N_2_ atmosphere. The formed bar was cut and grinded to approximately 2 mm grain size in a cutting mill (Fritsch Pulverisette, Fritsch GmbH, Idar-Oberstein, Germany). In the second stage, polymer fibers were melt spun and knitted as previously [19] except for PBS and PLA + 5 wt% PTMC fibers, which were spinned using a Fourné laboratory drawing line (Fourné Polymertechnik GmbH, Alfter-Impekoven, Germany) due to the difficulties with the fiber durability. The different methods and the differences in the material properties caused some variation in the filament thickness (ranging from 50 to 120 *μ*m). A representative image of a knitted scaffold is presented in Figure 1. The scaffolds were gamma-sterilized with a 25 kGy dose. The polymer proportions of the blends are presented in Table 2.

### 2.3. Material Characterization

Degradation of the fibers was determined in hydrolysis at 37°C in phosphate buffer solution according to ISO 15814. The samples were collected at 0, 1, 2, 3, and 4 week time points. The inherent viscosities of the samples were measured by viscometric analysis (Lauda PSV1, Lauda-Königshofen, Germany), and the thermal properties were studied with differential scanning calorimetry (DSC) with 20°C/min heating rate (Q 1000, TA Instruments, USA). For DSC, approximately 5 mg samples were placed into standard (not hermetic) aluminum DSC cups and the heating range was −50…+200°C. Two heating cycles were used, and the melt enthalpy was obtained from the first heating round. The % crystallinity of the polymer materials was calculated as follows: *χ* = 100^∗^(Δ*H*_m_)/(Δ*H*_lit_), where Δ*H*_m_ is the measured melt enthalpy of the sample material and Δ*H*_lit_ is the melting enthalpy of the 100% crystalline polymer material [20]. The following Δ*H*_lit_ values were used in the calculations: PLA 96 J/g [21], PBS 110.3 J/g [20], and PCL 139 J/g [22]. As PTMC is a fully amorphous material, its melting peak could not be detected and thus its % crystallinity was considered 0. In case of the blends, the % crystallinity of each component was summed up to give the crystallinity of the blend, according to the following formula:

(1) $$\begin{array}{c} \chi_{tot}=\left(\frac{{\Delta H}_{m,component1}}{{\Delta H}_{lit,component1}}+\frac{{\Delta H}_{m,component2}}{{\Delta H}_{lit,component2}}\right)\times 100\%. \end{array}$$

The tensile strength of the four-filament fiber was determined using a universal testing machine with a 500 N load cell (Instron 4411, Instron, Buckinghamshire, UK). 50 mm specimen gauge length was used with 30 mm/min testing speed. Before the testing, the fibers were rinsed three times with distilled water and gently wiped dry with cellulose paper.

Scaffold porosities were analyzed with X-ray microtomography imaging device Zeiss Xradia MicroXCT-400 (Zeiss, Pleasanton, USA). The field of view was cylindrical, 5.8 mm wide and high, from the center of the sample. The source voltage was selected to 80 kV, source current to 125 *μ*A, and the voxel size to 2.94 *μ*m. Porosities were calculated from the reconstructed image stacks with Avizo 9.3.0 (FEI, Hillsboro, Oregon, USA) by using manual thresholding in the segmentation procedure.

### 2.4. Adipose Stem Cell Isolation, Expansion, and Culture

The isolation of hASCs was conducted using a mechanical and enzymatic procedure described previously [23, 24]. The isolated hASCs were maintained in T-75 polystyrene flasks (Nunc, Thermo Fisher Scientific, Waltham, MA, USA) in DMEM/F-12 (Life Technologies, Thermo Fisher Scientific) supplemented with 5% human serum (PAA Laboratories, GE Healthcare, Little Chalfont, Buckinghamshire, United Kingdom), 1% L-glutamine (GlutaMAX I, Life Technologies, Thermo Fisher Scientific), and 1% antibiotics (100 U/ml penicillin and 0.1 mg/ml streptomycin, BioWhittaker, Lonza, Basel, Switzerland). The hASCs used in the experiments had strong expression (>95%) for surface proteins CD73, CD90, and CD105; low expression (<2%) of CD3, CD11a, CD14, CD19, CD45, CD80, CD86, and HLA-DR; and moderate expression (<20%) of CD34 and CD54, thus verifying the mesenchymal origin of the cells. Human ASC donor lines used were in passages 2–4.

For all the experiments, 100,000 cells/scaffold were seeded in a volume of 100 *μ*l. After 3 h of attachment, 1.5 ml medium was added to each well (24-well plates, Nunc). The following day, osteogenic medium (OM; maintenance medium supplemented with 10 mM *β*-glycerophosphate, 250 *μ*M L-ascorbic acid 2-phosphate, and 5 nM dexamethasone; all from Sigma-Aldrich, St. Louis, MO, USA) was given to the cells, and the rest of the culture was conducted in OM. After 11 days of culture, the scaffolds were transferred to bigger wells (12-well plates, Nunc) and the culture was continued in a volume of 3 ml/well in order to avoid excessive pH rise and to provide enough nutrients for the increased amount of cells. During the experiments, the medium was changed twice a week.

### 2.5. Cell Viability

Cell viability in the various culturing conditions at 14 d was analyzed by Live/Dead staining (Invitrogen, Thermo Fisher Scientific) as described previously [24]. Briefly, cells were incubated in working solution containing 0.25 *μ*M EthD-1 and 0.5 *μ*M calcein-AM for 30 min. After the incubation, samples were imaged immediately with fluorescence microscope (IX51, Olympus, Tokyo, Japan).

### 2.6. Cell Proliferation

Cell proliferation was studied by determining the DNA amount using a CyQUANT Cell Proliferation Assay kit (Invitrogen, Thermo Fisher Scientific), according to the manufacturer's protocol. Briefly, at 7 d and 14 d time points cells were lysed with 0.1% Triton-X 100 (Sigma-Aldrich) buffer. After two freeze-thaw cycles (−70°C), three parallel 20 *μ*l samples of each lysate were pipetted to a 96-well plate and mixed with 180 *μ*l working solution. The fluorescence at 480/520 nm was measured with a Victor 1420 Multilabel counter (Wallac, Turku, Finland).

### 2.7. Phalloidin Staining

In order to visualize the actin cytoskeleton of the hASCs grown on the different materials, actin cytoskeleton was stained with phalloidin after 7 days of culture. Briefly, the cells were fixed and permeabilized with 0.2% Triton-X 100 in 4% paraformaldehyde (PFA; Sigma-Aldrich) for 15 min at room temperature (RT). The samples were blocked with 1% bovine serum albumin for 1 h, and Alexa Fluor™ 488 Phalloidin (Molecular Probes, Thermo Fisher Scientific; diluted in blocking solution 1 : 200) was incubated for 45 min at RT. In order to stain the nuclei, 4′,6-diamidino-2-phenylindole (DAPI; Molecular Probes, Thermo Fisher Scientific; dilution 1 : 2000) was applied in the last washes. The samples were imaged with a laser scanning confocal microscope Zeiss LSM 780 inverted Zeiss Cell Observer.Z1 body using a Zeiss LD LCI Plan-Apochromat 25x (numerical aperture = 0.8) water immersion objective (Zeiss, Oberkochen, Germany). 488 nm and 405 nm laser lines were used to excite the fluorophores. Image stacks of 100 slices/50 *μ*m in range were captured with a voxel size of 119 nm in the *x* and *y* dimensions and 500 nm in the *z* dimension. The Alexa Fluor 488 fluorescence was collected using a 410–495 nm filter and DAPI with a 495–630 nm filter. The pinhole was adjusted to 1 Airy unit. Image deconvolution was performed with Huygens Essential (Scientific Volume Imaging, Hilversum, Netherlands) with a signal-to-noise ratio of 5, quality threshold of 0.001, and 200 as the maximum number of iterations.

### 2.8. Alkaline Phosphatase Activity

ALP activity was determined quantitatively after 7 d and 14 d of culture, as previously described [24, 25]. The activity was analyzed from the same Triton-X 100 lysates as the DNA amount. In short, 20 *μ*l of each lysate was pipetted in three parallel samples into the wells of a MicroAmp™ Optical 96-well plate (Applied Biosystems, Thermo Fisher Scientific). 90 *μ*l of working solution containing 1 : 1 stock substrate solution (*p*-nitrophenol phosphate) (Sigma-Aldrich) and 1.5 M alkaline buffer solution (2-amino-2-methyl propanol) (Sigma-Aldrich) was added to each well and, after 15 min incubation at +37°C 50 *μ*l of 1 M NaOH (Sigma-Aldrich), was added to the wells to stop the reaction. Finally, the absorbances were measured with a Victor 1420 Multilabel counter (Wallac) at 405 nm.

### 2.9. Quantitative Real-Time PCR

The relative expression of osteogenic marker genes was studied at 7 d and 14 d by quantitative real-time reverse transcription polymerase chain reaction (qRT-PCR) as described previously [26]. In short, the total messenger RNA (mRNA) was isolated from the samples using NucleoSpin RNA II kit (Macherey-Nagel, Düren, Germany) after which the isolated mRNA was reverse transcribed to cDNA with the High-Capacity cDNA Reverse Transcriptase Kit (Applied Biosystems, Thermo Fisher Scientific). The expressions of osteogenic marker genes *DLX5*, *OSTERIX*, and *RUNX2a* were analyzed, and the data was normalized to the expression of housekeeping gene *RPLP0* (*human acidic ribosomal phosphoprotein P0*). In the calculation of relative expressions, a previously described mathematical model was used [27]. The sequences of the primers (Oligomer Oy, Helsinki, Finland) and the accession numbers of the genes studied can be found from our previous publication [25]. The qRT-PCR mixture contained 50 ng cDNA, 300 nM forward and reverse primers, and Power SYBR® Green PCR Master Mix (Applied Biosystems, Thermo Fisher Scientific). The reactions were conducted and monitored with ABI Prism 7000 Sequence Detection System (Applied Biosystems, Thermo Fisher Scientific) with initial enzyme activation at 95°C for 10 min, followed by 45 cycles at 95°C/15 s and 60°C/60 s.

### 2.10. Mineralization

Mineralization at 27 d was assessed by Alizarin red S staining following a previously described protocol [26]. Briefly, cells were fixed with 4% PFA for 35 min at RT and stained with 2% Alizarin red S (pH 4.1–4.3; Sigma-Aldrich) solution for 10 min at RT. The excess color was washed away, and the dye was extracted with 100 mM cetylpyridinium chloride (Sigma-Aldrich). After 3.5 h hours of extraction, the absorbances were measured with Victor 1420 Multilabel counter (Wallac) at 544 nm.

### 2.11. Statistical Analyses

Statistical analyses were performed with SPSS Statistics version 22 (IBM, Armonk, NY, USA). All the quantitative data is presented as mean and standard deviation (SD). The statistical significances were evaluated with nonparametric statistics using the Mann–Whitney test. The resulting *p* values were corrected using Bonferroni adjustment based on the number of the planned comparisons. The result was considered statistically significant when the adjusted *p* value < 0.05. All the conducted comparisons and the corresponding *p* values are presented in Supplementary Tables 1S and 2S.

## 3. Results

### 3.1. Material Characterization

**Figure 2:**
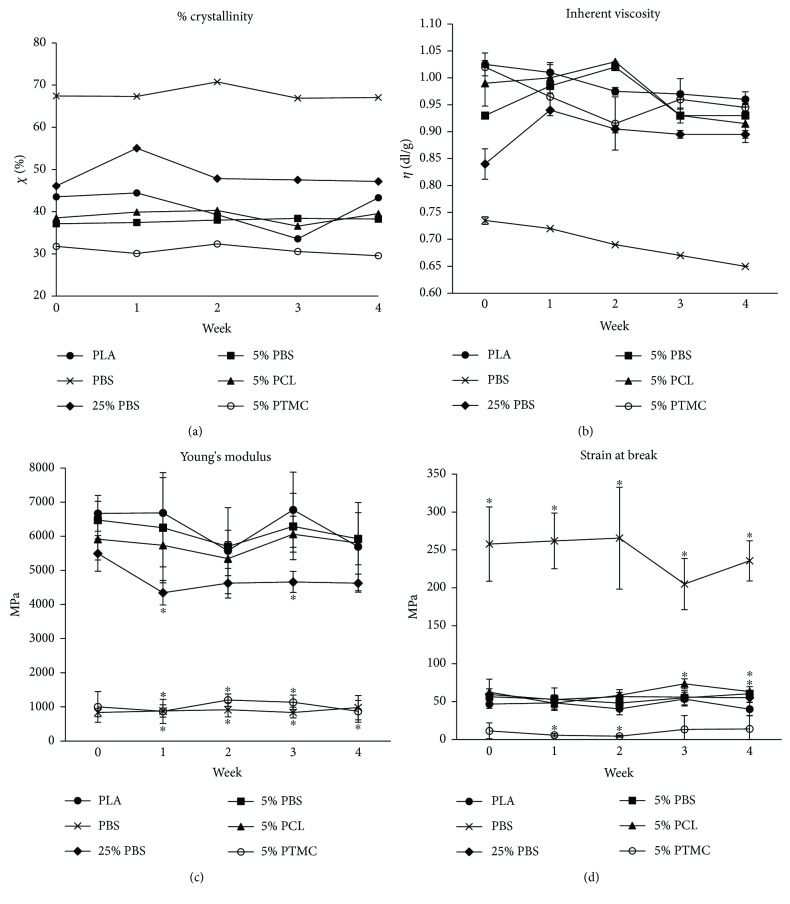
Material characterization. (a) Crystallinity of the materials after 0, 1, 2, 3, and 4 weeks of hydrolysis. (b) Inherent viscosity after 0, 1, 2, 3, and 4 weeks of hydrolysis; *n* = 1‐2. (c) Toughness of the materials after 0, 1, 2, 3, and 4 weeks of hydrolysis; *n* = 3–5. (d) Strain at break after 0, 1, 2, 3, and 4 weeks of hydrolysis; *n* = 4‐5. *p* < 0.05 between the indicated material (^∗^) and PLA at the same time point.

Melt enthalpy (the energy needed to melt a material) can be used to compare the rate of crystallinity of different materials. In this case, pure PBS had the highest crystallinity (Figure 2(a)). The blending has also an effect on end material crystallinity depending on the amounts of materials blended and the crystallinities of the raw materials. PBS and PCL were more crystalline than PLA whereas PTMC was amorphous. The crystallinities of the polymer materials remained the same during the four-week hydrolysis.

Inherent viscosity can be used to describe the degradation behavior of a polymer material during hydrolysis. The more the material degrades, the lower the inherent viscosity. Here, the materials have started to degrade but since the four-week period is so short, the viscosity values did not markedly change (Figure 2(b)).

Young's modulus describes the toughness of the material, and the strain at break tells about the material's ability to deform under force. PLA-based blends excluding 5% PTMC were tougher when compared to PBS (Figures 2(c) and 2(d)). Because of the low miscibility of the materials, 5% PTMC had low Young's modulus and strain at break. PTMC formed low-strength bumps to the fiber during the fiber spinning, making it fragile. The high strain of PBS can be explained with the high crystallinity: when force is applied on the material, the crystallites start to untangle enabling the fibers to stretch.

Regarding the porosity of the knitted and rolled 3D scaffolds, all the scaffolds had open pores and a porosity ranging from 55.5% to 76.3%. The porosities for each of the material are presented in Table 3. The porosity increased slightly with increasing PBS content, but otherwise, the differences in the porosities were relatively small.

### 3.2. Cell Viability, Proliferation, and Attachment on the Scaffolds

**Figure 3:**
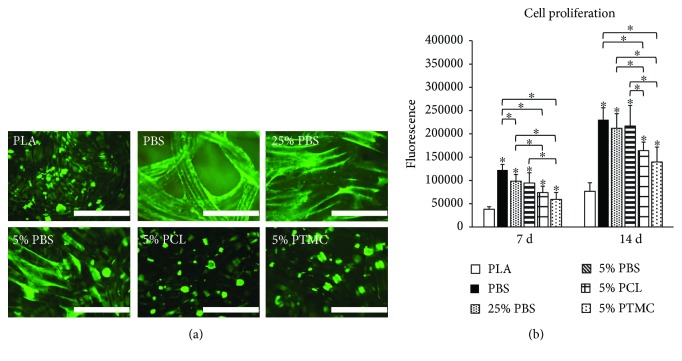
Human ASC viability and proliferation on knitted 3D scaffolds. (a) Human ASC viability at 14 d analyzed by Live/dead staining. Living cells are stained green and dead cells red. Scale bars: 1.0 mm. (b) Human ASC proliferation at 7 d and 14 d as determined by the CyQUANT cell proliferation assay; *n* = 12. *p* < 0.05 between the indicated material (^∗^) and PLA at the same time point (unless otherwise indicated).

To assess the viability of the hASCs on the knitted 3D scaffolds, Live/Dead staining was conducted after 14 days of culture. As seen from Figure 3(a), all the materials supported cell viability since no dead cells could be detected. However, clear differences in the cell arrangement were observed. On PLA, 5% PCL, and 5% PTMC, cells formed large clusters, whereas on PBS-containing materials, cells were able to align along the fibers. The ability to align was the most prevalent with pure PBS and decreased with decreasing PBS content. With respect to cell proliferation, all the materials increased the proliferation significantly at both time points when compared to the control PLA (Figure 3(b)). Specifically, the strongest proliferative response was detected on the PBS containing materials and was not dependent on the PBS content.

**Figure 4:**
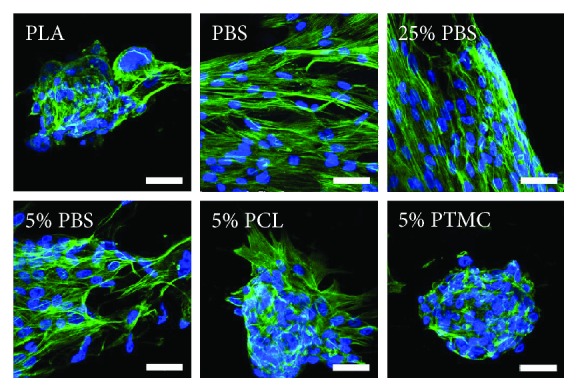
Cytoskeletal organization of hASCs on knitted 3D scaffolds. Phalloidin staining of the actin cytoskeleton (green). Nuclei were stained with DAPI (blue). Scale bars: 50 *μ*m.

The attachment of the hASCs on the scaffolds was evaluated with phalloidin staining of the actin cytoskeleton after 7 days of culture. As seen from Figure 4, actin staining was well in line with the observations made from the Live/Dead analysis. The actin cytoskeleton was oriented parallel to the fibers on PBS and to some extent also on 25% and 5% PBS, but on PLA, 5% PCL, and 5% PTMC, the cells formed large clusters with no signs of proper alignment along the fibers.

In order to further assess the cell attachment and growth inside the scaffolds, histological samples were prepared and stained with HE staining after 27 days of culture (see supplementary data). Even though no bone-like tissue was detected yet at this time point, the cell ingrowth was still evidently best in PBS materials, whereas in PLA, 5% PCL, and 5% PTMC samples, only cell clusters, similar to those observed in Live/Dead and phalloidin stainings, were detected (Supplementary Figure 1S).

### 3.3. Alkaline Phosphatase Activity and the Expression of Osteogenic Marker Genes

**Figure 5:**
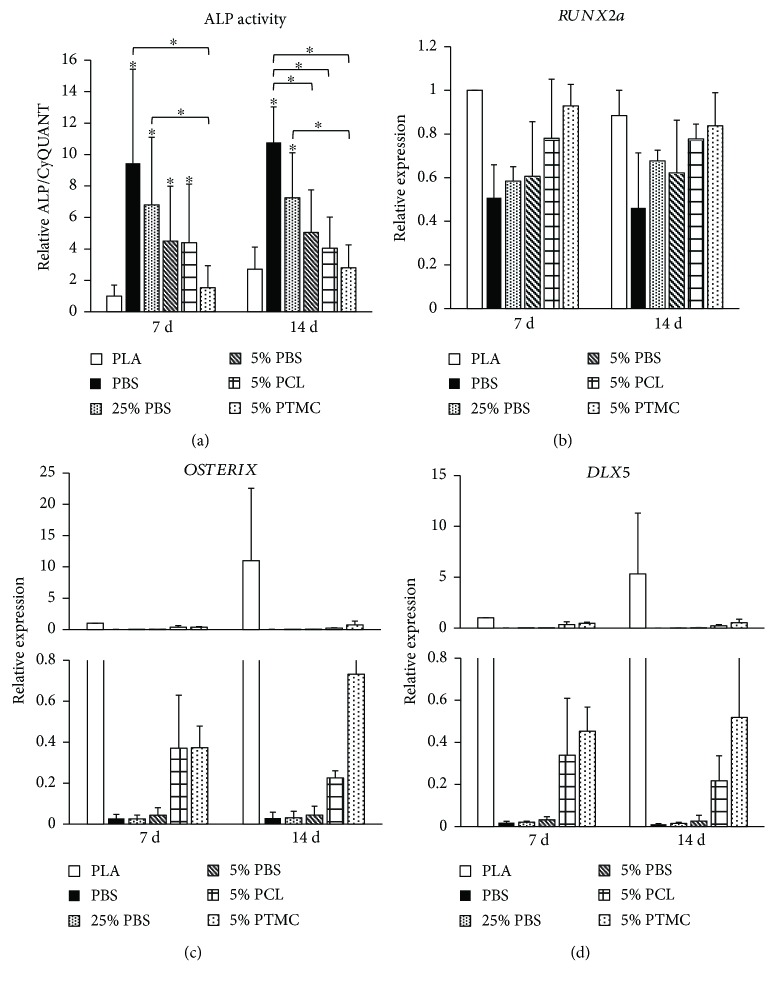
Alkaline phosphatase activity and the expression of osteogenic marker genes on knitted 3D scaffolds. (a) Alkaline phosphatase activity normalized with CyQUANT cell proliferation results at 7 d and 14 d; *n* = 12. *p* < 0.05 between the indicated material (^∗^) and PLA at the same time point (unless otherwise indicated). The results are relative to the 7 d PLA sample. (b) *RUNX2a* expression at 7 d and 14 d; *n* = 4. The results are relative to the 7 d PLA sample. (c) *OSTERIX* expression at 7 d and 14 d; *n* = 4. The results are relative to the 7 d PLA sample. (d) *DLX5* expression at 7 d and 14 d; *n* = 4. The results are relative to the 7 d PLA sample.

In order to evaluate the early stages of osteogenic differentiation of hASCs cultured in the knitted 3D scaffolds, quantitative ALP activity and the expression of osteogenic marker genes *RUNX2a*, *OSTERIX*, and *DLX5* were assessed after 7 and 14 days of culture. Initially, at the 7 d time point, all the PBS materials as well as 5% PCL induced significantly higher ALP activities than the control PLA did (Figure 5(a)). At 14 d, however, the differences had narrowed and only PBS and 25% PBS stimulated significantly higher ALP activities than did PLA. PBS was clearly the strongest inducer of ALP activity, and this ability declined with the decreasing PBS content. In contrast to the ALP activity results, the performance of the PBS materials in supporting osteogenic marker gene expression was worst of all the materials studied (Figures 5(b)–5(d)). Unexpectedly, the highest gene expression levels were measured from the PLA sample, followed by 5% PTMC and 5% PCL. In case of *RUNX2a* expression (Figure 5(b)), the differences between the samples were only moderate, but with *OSTERIX* and *DLX5*, there was a considerable drop in the expression in the PBS-containing samples (Figures 5(c) and 5(d)).

### 3.4. Mineralization

**Figure 6:**
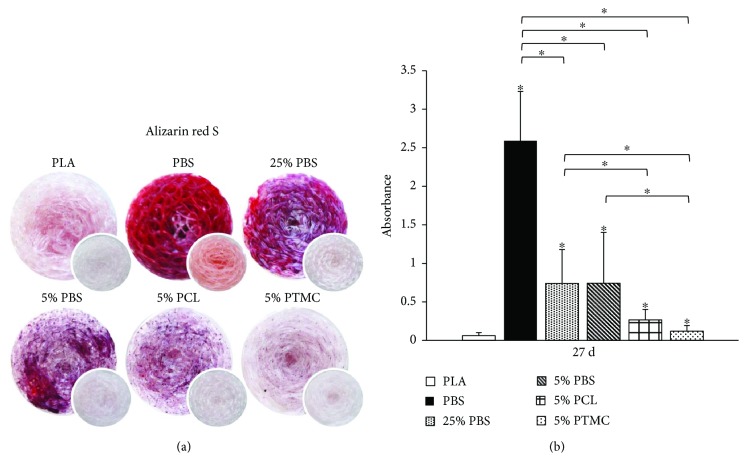
Mineralization on knitted 3D scaffolds. (a) Alizarin red S staining of the scaffolds after 27 d of culture. CaP mineral is stained red. The smaller images represent blank samples (no cells). Each image shows the whole scaffold (diameter 10 mm). (b) Quantification of Alizarin red S staining at the 27 d time point; *n* = 9. *p* < 0.05 between the indicated material (^∗^) and PLA at the same time point (unless otherwise indicated).

The later stages of osteogenic differentiation were analyzed with Alizarin red S mineralization staining after 27 days of culture. As seen in Figure 6(a), a proper mineralization, as evidenced by the red-stained CaP, was detected only in the pure PBS sample. Moreover, traces of mineral formation were also visible in 25% PBS and 5% PBS samples, whereas with other materials no indications of mineral deposition could be detected. These observations were also reflected to the quantitative results (Figure 6(b)), which show that PBS was significantly the strongest inducer of mineralization. Furthermore, 25% PBS and 5% PBS supported mineral formation significantly better than the rest of the materials.

## 4. Discussion

Since the 1990s, PBS has been widely exploited as biodegradable packaging material, but only quite recently has it started to raise interest in the field of regenerative medicine due to its many favorable properties. The accumulating evidence about the good performance of PBS in biomedical approaches prompted us to utilize PBS and PBS-PLA blends in textile-based manufacturing of knitted 3D scaffolds for the evaluation of hASC attachment, proliferation, and osteogenic differentiation in *in vitro* culture.

Our results demonstrate that cell attachment and spreading were drastically improved on PBS-PLA blends and especially on pure PBS when compared to pure PLA. However, the material characterizations conducted during the four-week hydrolysis did not give any clear indications for why cells seemed to prefer PBS. Specifically, no great changes in the material properties were observed during this relatively short hydrolysis time. Still, we were determined to restrict the hydrolysis period to four weeks since this corresponded to the duration of the cell culture and thus to the changes in the material properties the cells experienced during the *in vitro* experiment. Regarding differences between the different materials, PBS was clearly the most crystalline material, which in part explains the high elongation at break values, but this cannot be directly linked to the cell behavior without further research. It has been previously shown that PBS is more hydrophilic than PDLLA [8], which might explain the better cell attachment since it typically favors hydrophilic surfaces over hydrophobic. The potentially too high hydrophobicity of PBS has caused some concern, which has led many studies to implement different surface treatments (hydrolysis, etching, plasma treatment, UV oxidation, etc.) to increase the hydrophilicity and thus better facilitate the cell attachment [9–11, 28]. However, our results suggest that hydrophobicity is not a problem and no surface treatment of PBS is needed for the cells to attach and spread on the material surface. Interestingly, a distinctly patterned roughness profile was observed on the surface of PBS fibers with SEM (Supplementary Figure 2S), possibly reflecting a fiber relaxation phenomenon. Such roughness, not detected on the other materials, might also favor the cell attachment and partially explain the good results obtained with PBS. Overall, the superior cell attachment on PBS is a clear advantage over PLA, which cannot support proper cell attachment without additional surface manipulations.

Well in line with the cell spreading along the fibers, osteogenic differentiation of hASCs was also clearly enhanced on PBS and PLA-PBS blends compared to PLA, PLA-PCL, and PLA-PTMC on which the cells retained a rounded morphology. It has been frequently reported that the ability of cells to spread or elongate is required for the commitment of osteogenic fate, whereas rounded morphology prohibits osteogenesis and guides stem cells towards other directions, such as adipogenesis [29–32]. The significantly enhanced ALP activity as well as mineralization on cell spreading promoting PBS scaffolds clearly supports these observations. Unexpectedly, the gene expression profiles of osteogenic marker genes did not follow this scheme; the expression of *RUNX2a*, *OSTERIX*, and *DLX5* were constantly the lowest on PBS and PLA-PBS blends, with pure PLA showing the highest expression levels. It is possible that the time frame of the qRT-PCR analysis did not reveal the expression peaks of these genes on PBS materials. However, our preliminary 4 d gene expression data with a very similar pattern as in the 7 d and 14 d data (data not shown) does not support this conclusion. Recently, there has been some evidence that culturing MSCs in spheroids might increase their differentiation potential, including differentiation towards osteogenic fate [33, 34]. Therefore, the cell cluster formation on PLA, PLA-PCL, and PLA-PTMC, likely as a result of poor attachment, might have triggered an osteogenic program in the hASCs. However, based on negligible ALP activity and mineralization on these materials, the osteogenesis did not proceed to the later stages, which might be related to the small overall cell amount. On PBS materials, on the other hand, the large cell density might have comprised undifferentiated areas pulling down the normalized gene expression values. Still, due to the high cell density, large areas were fully committed towards bone as observed.

Mesenchymal stem cells have been widely accepted as an excellent cell type for regenerative medicine applications due to their various advantageous properties (e.g., ease of isolation, multipotency, and immunomodulatory properties) [35]. However, hMSC quality and characteristics are known to be affected by several features, including donor age, gender, body mass index, and, in case of hASCs, the adipose tissue harvest site [36–40]. This donor-to-donor variation was also reflected to our late osteogenic differentiation results; out of the five donor hASC lines studied, three showed excessive mineralization on PBS, whereas two donor lines proved to be incapable of mineral production in all the materials as indicated in Supplementary Figure 3S. We have previously demonstrated that in response to BMP-2, hASCs from some donors favor osteogenic fate whereas other donor cell lines tend to commit towards adipocytes [41]. Moreover, hASCs from different donors showed variable tendency for mineralization even in unsupplemented OM culture. These observations are well in line with the results of the present study, which further emphasizes the MSC donor-to-donor variability as a critical factor to take into account when evaluating the therapeutic potential of TE structures.

It has been previously demonstrated that upon subcutaneous implantation in rats, discs of PBS and PLA-PBS blend (50/50 wt%) induce only a mild inflammation and foreign body reaction, and the fibrous capsule thickness was the smallest with PLA-PBS blend when compared to PBS and PLA [15]. Moreover, in mouse, critical-sized calvarial defect model 3D porous PBS-chitosan blend (50/50 wt%) scaffolds demonstrated good integration with the surrounding tissues and enhanced bone formation, which was even more evident with hBMSC-seeded scaffolds [13]. These studies demonstrate that PBS is well tolerated *in vivo* and combined with chitosan is able to support bone formation. However, in future more evidence is needed about the *in vivo* performance of PBS as such and in comparison with PLA and other similar polymers.

## 5. Conclusions

In conclusion, pure PBS was observed to have the highest crystallinity and strain at break compared to the tougher PLA and PLA blends. However, no degradation occurred during the 4-week hydrolysis period in either of the materials. Our results revealed clearly enhanced cell attachment, proliferation, and osteogenic differentiation of hASCs on knitted 3D scaffolds of PBS and PLA-PBS blends when compared to scaffolds of PLA, as well as PLA-PCL and PLA-PTMC blends. The beneficial effects of PBS were observed to be dependent on the PBS content, with pure PBS eliciting the most favorable cell responses. Being a cheap, easily processable, biodegradable, and biocompatible cell growth and differentiation supporting material, PBS possesses great promise to be more widely used as a scaffolding material in TE applications. Remarkably, it outperformed the traditionally used PLA, which further encourages a more thorough evaluation and characterization of PBS as well as PBS-based blends and composites for the purposes of regenerative medicine.

## Figures and Tables

**Table 1:** Inherent viscosities of the polymers.

Polymer	Inherent viscosity (dl/g)
PLA	2.18
PCL	1.06
PBS	1.07
PTMC	3.07

**Table 2:** Polymer proportions of the blends used in this study.

Material	PLA (weight %)	Other component (weight %)
PLA	100	0
PBS	0	100
PLA + 25 wt% PBS	75	25
PLA + 5 wt% PBS	95	5
PLA + 5 wt% PCL	95	5
PLA + 5 wt% PTMC	95	5

**Table 3:** Scaffold porosities (*n* = 3).

Material	Average porosity ± standard deviation (%)
PLA	62.6 ± 2.6
PBS	76.3 ± 4.3
25% PBS	65.3 ± 6.4
5% PBS	58.0 ± 3.0
5% PCL	55.5 ± 5.1
5% PTMC	57.9 ± 7.4

## Supplementary Materials

Supplementary Materials Histological analysis of the 3D scaffolds after 27d *in vitro* culture. Supplementary Figure 1S: histological cross sections were stained with hematoxylin and eosin. Supplementary Figure 2S: SEM images of cell-containing and cell-free scaffolds after 27 d *in vitro* culture are presented. Supplementary Figure 3S: the 27 d mineralization result of the hASC donor lines, which did not produce mineral during the culture. Supplementary Tables 1S and 2S: the exact Bonferroni-corrected *p* values obtained with the nonparametric Mann–Whitney test for the quantitative data.
