# Supplementary material for: Knitted 3D Scaffolds of Polybutylene Succinate Support Human Mesenchymal Stem Cell Growth and Osteogenesis
Source: Stem Cells Int. 2018 May 7;2018:5928935. doi: 10.1155/2018/5928935 (PMC5964421; doi:10.1155/2018/5928935)
Supplement: Supplementary Materials — Histological analysis of the 3D scaffolds after 27d in vitro culture. Supplementary Figure 1S: histological cross sections were stained with hematoxylin and eosin. Supplementary Figure 2S: SEM images of cell-containing and cell-free scaffolds after 27 d in vitro culture are presented. Supplementary Figure 3S: the 27 d mineralization result of the hASC donor lines, which did not produce mineral during the culture. Supplementary Tables 1S and 2S: the exact Bonferroni-corrected p values obtained with the nonparametric Mann–Whitney test for the quantitative data. [file 5928935.f1.pdf]

SUPPLEMENTARY MATERIALS

Materials and methods

Histological analysis

After 27 days of culture the scaffolds were fixed with 4% paraformaldehyde for 1 h at room temperature, embedded in paraffin and sectioned at 5 μm thickness for histologic analyses. Hematoxylin and Eosin (HE) staining was conducted to examine the tissue formation within the scaffolds. The samples were imaged with Zeiss Axio Scope.A1 microscope.

Scanning electron microscopy

For scanning electron microscopy (SEM) the cells were cultured for 27 days in the scaffolds, after which they were fixed with 5 % glutaraldehyde (Sigma-Aldrich) for 1 h at room temperature. The glutaraldehyde solution was replaced with water for 30 min, followed by dehydration with ascending series of ethanol (10 %, 20 %, 40 %, 60 %, 80 % and 99.5 % ethanol; 10 min each). The same treatment was also conducted for cell-free blank samples which were incubated in the medium for 27 days prior to the SEM analysis. The fixed samples were dried in vacuum overnight and sputter-coated with gold (Edwards S150 Sputter Coater). The samples were then imaged with SEM (Philips XL-30, Philips, Amsterdam, the Netherlands).

Results

Histological analysis

In order to assess the tissue formation inside the scaffolds during the 27d culturing period, histological samples were prepared and stained with HE staining. Even though no bone-like tissue was detected yet at this time point, the cell ingrowth was still evidently best in PBS materials, whereas in PLA, 5% PCL and 5% PTMC samples only cell clusters were detected (Supplementary Figure 1S).

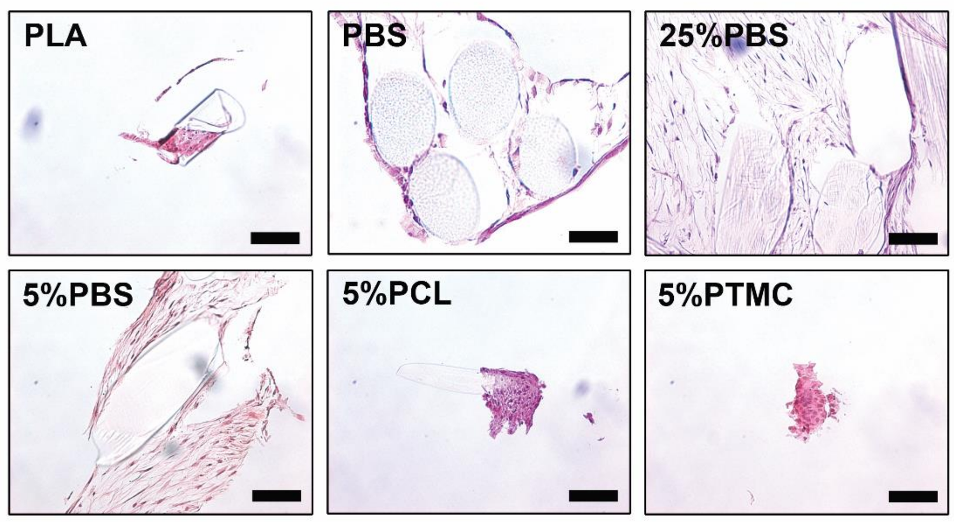

Supplementary Figure 1S. Tissue formation in the knitted 3D scaffolds. Histological cross-sections stained with HE staining. Cells were cultured in the scaffolds for 27d. Scale bars 100 µm.

Scanning electron microscopy

To further evaluate the fiber topography as well as the cell attachment on the scaffolds, SEM analysis was conducted after 27 days of culture. In Supplementary Figure 2SA blank scaffolds (not containing cells) are depicted. Unlike the other fibers the surface of PBS fibers shows distinct topographical features and nano/micro scale roughness, possibly reflective of a fiber relaxation phenomenon. With respect to cell amount and attachment, it was also apparent in SEM images that on PLA, 5% PBS, 5% PCL and 5% PTMC the cells had problems to attach properly and thus formed sparsely arranged cell clusters (Supplementary Figure 2SB). On PBS and 25% PBS, on the other hand, the cells had proliferated extensively and fully covered both fibers and inter-fiber spaces.

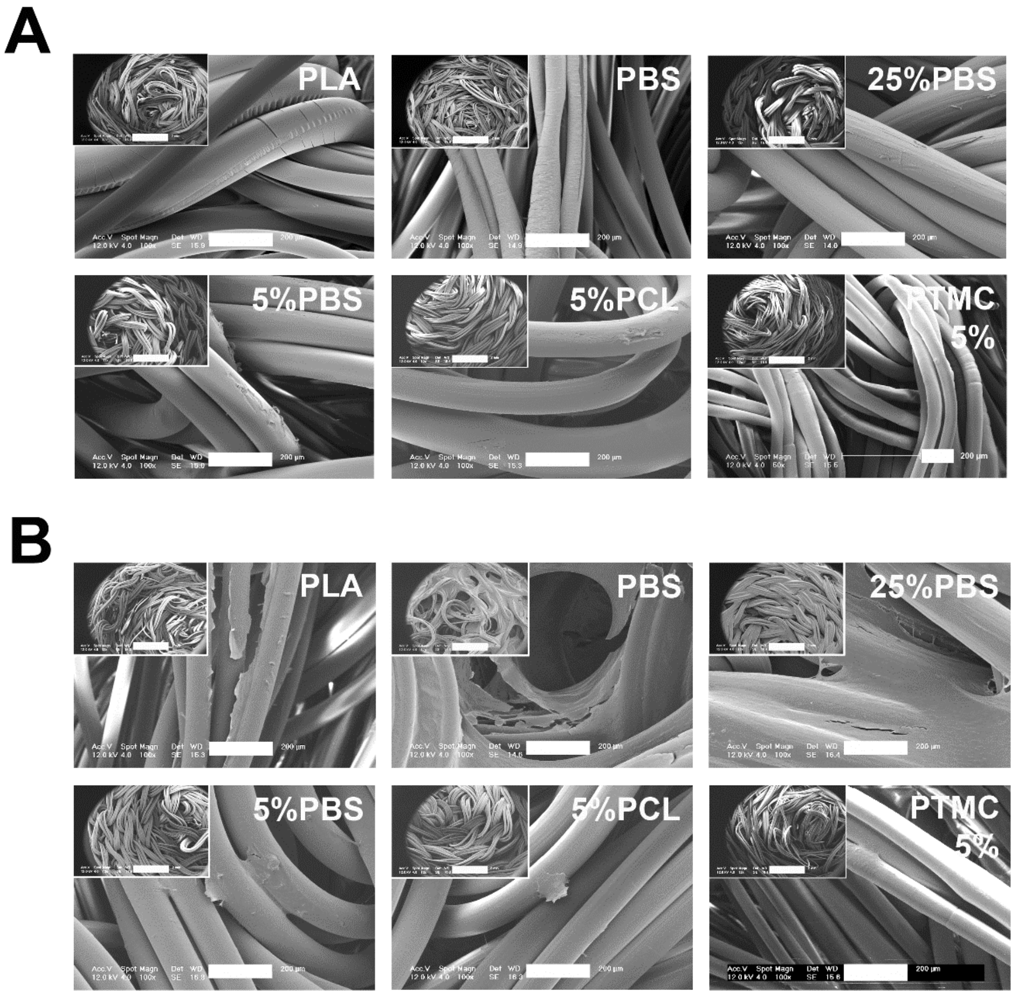

Supplementary Figure 2S. Scanning electron microscopy of the knitted 3D scaffolds. **A.** SEM images of blank samples (no cells) after 27d incubation in the cell culture medium. Scale bars 200 µm (scales of the smaller images 2 mm). **B.** SEM images of cell-containing samples after 27d of culture. Scale bars 200 µm (scales of the smaller images 2 mm).

Alizarin red S staining of non-mineralizing donor hASCs

Out of the five hASC donor lines studied, hASCs from three donors were able to form mineral in the PBS-containing scaffolds as reported in the main article. However, two of the donor lines did not show any signs of mineralization after the 27d culturing period in OM, as seen in Supplementary Figure 3SA. The deep purple stain seen in the figure is caused by the cell-related background and does not indicate mineral formation, which is detected as bright red stain. Therefore, even though some statistical differences were observed in the quantified results (Supplementary Figure 3SB) this should not be incorrectly interpreted as enhanced mineralization. In general, the quantified

absorbance values were clearly smaller compared to the values of actually mineralized samples (Figure 6).

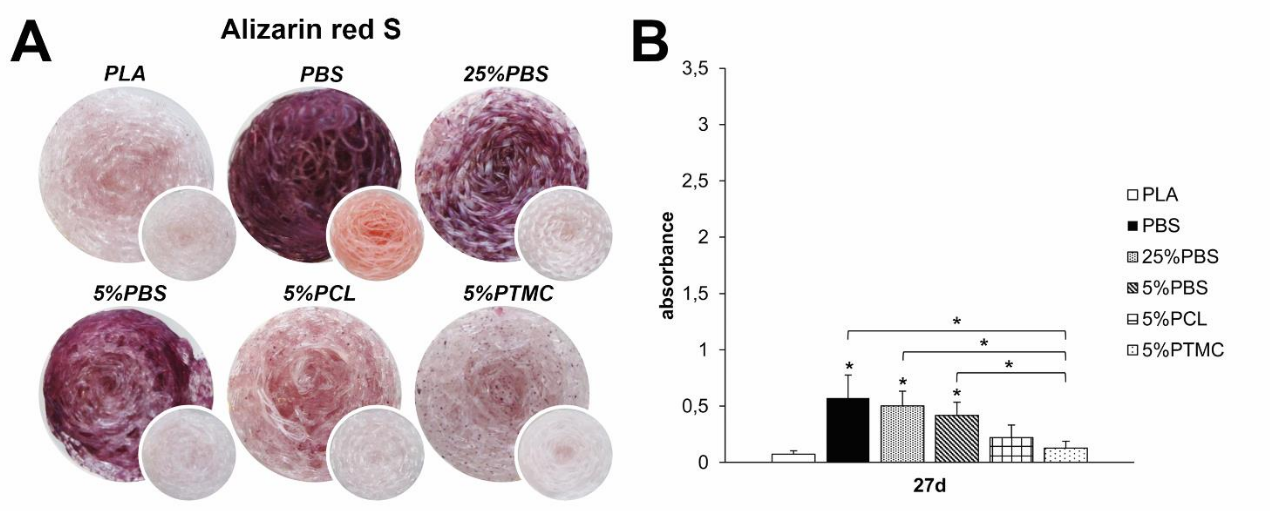

Supplementary Figure 3S. Alizarin red S staining of non-mineralizing ASC donor cells on knitted 3D scaffolds. **A.** Alizarin red S staining of the scaffolds after 27d of culture. CaP mineral is stained red. The smaller images represent blank samples (no cells). Each image shows the whole scaffold (diameter 10 mm). **B.** Quantification of the Alizarin red S staining at 27d time point. n=6. $p<0.05$ between the indicated material (*) and PLA at the same time point (unless otherwise indicated).

Supplementary Table S1. The exact Bonferroni corrected p-values obtained with the non-parametric Mann-Whitney test for cell data. The following Bonferroni coefficients (the number of comparisons) were used: 36 for CyQUANT (n=12), 36 for qALP/CyQUANT (n=12), 15 for Alizarin red S (n=9), and 15 for supplemental Alizarin red S (n=6). p-values<0.05 are presented in bold.

**CyQUANT 7d**		**CyQUANT 14d**		**CyQUANT 7d vs. 14d**	
comparison	***p-value***	***comparison***	***p-value***	***comparison***	***p-value***
PLA vs. PBS	**2.66E-05**	PLA vs. PBS	**2.66E-05**	PLA	**2.66E-05**
PLA vs. 25% PBS	**2.66E-05**	PLA vs. 25% PBS	**2.66E-05**	PBS	**2.66E-05**
PLA vs 5% PBS	**2.66E-05**	PLA vs 5% PBS	**2.66E-05**	25% PBS	**2.66E-05**
PLA vs. 5% PCL	**2.66E-05**	PLA vs. 5% PCL	**2.66E-05**	5% PBS	**2.66E-05**
PLA vs. 5% PTMC	**5.04E-04**	PLA vs. 5% PTMC	**3.24E-04**	5% PCL	**2.66E-05**
PBS vs. 25% PBS	**1.80E-03**	PBS vs. 25% PBS	1.00	5% PTMC	**2.66E-05**
PBS vs. 5% PBS	1.31E-01	PBS vs. 5% PBS	1.00		
PBS vs. 5% PCL	**2.66E-05**	PBS vs. 5% PCL	**3.60E-05**		
PBS vs. 5% PTMC	**2.66E-05**	PBS vs. 5% PTMC	**1.08E-04**		
25% PBS vs. 5% PBS	1.00	25% PBS vs. 5% PBS	1.00		
25% PBS vs. 5% PCL	**2.36E-02**	25% PBS vs. 5% PCL	**7.24E-03**		
25% PBS vs. 5% PTMC	**7.92E-04**	25% PBS vs. 5% PTMC	**2.59E-03**		
5% PBS vs. 5% PCL	8.71E-01	5% PBS vs. 5% PCL	**1.34E-02**		
5% PBS vs. 5% PTMC	**7,24E-03**	5% PBS vs. 5% PTMC	**3.71E-03**		
5% PCL vs. 5% PTMC	1.00	5% PCL vs. 5% PTMC	1.00		
**qALP/CyQUANT 7d**		**qALP/CyQUANT 14d**		**qALP/CyQUANT 7d vs. 14d**	
comparison	***p-value***	***comparison***	***p-value***	***comparison***	***p-value***
PLA vs. PBS	**2.66E-05**	PLA vs. PBS	**2.66E-05**	PLA	6.59E-02
PLA vs. 25% PBS	**3.60E-05**	PLA vs. 25% PBS	**7.24E-03**	PBS	1.00
PLA vs 5% PBS	**9.86E-03**	PLA vs 5% PBS	4.35E-01	25% PBS	1.00
PLA vs. 5% PCL	**1.79E-02**	PLA vs. 5% PCL	1.00	5% PBS	1.00
PLA vs. 5% PTMC	1.00	PLA vs. 5% PTMC	1.00	5% PCL	1.00
PBS vs. 25% PBS	1.00	PBS vs. 25% PBS	4.35E-01	5% PTMC	1.00
PBS vs. 5% PBS	5.22E-01	PBS vs. 5% PBS	**1.34E-02**		
PBS vs. 5% PCL	3.62E-01	PBS vs. 5% PCL	**2.66E-05**		
PBS vs. 5% PTMC	**1.80E-04**	PBS vs. 5% PTMC	**2.66E-05**		
25% PBS vs. 5% PBS	1.00	25% PBS vs. 5% PBS	1.00		
25% PBS vs. 5% PCL	1.00	25% PBS vs. 5% PCL	1.05E-01		
25% PBS vs. 5% PTMC	**2.59E-03**	25% PBS vs. 5% PTMC	**5.18E-03**		
5% PBS vs. 5% PCL	1.00	5% PBS vs. 5% PCL	1.00		
5% PBS vs. 5% PTMC	1.62E-01	5% PBS vs. 5% PTMC	1.00		
5% PCL vs. 5% PTMC	2.99E-01	5% PCL vs. 5% PTMC	1.00		
**Alizarin red S 27d**		**Alizarin red S 27d (Suppl.)**			
comparison	***p-value***	***comparison***	***p-value***		
PLA vs. PBS	**6.15E-04**	PLA vs. PBS	**3.25E-02**		
PLA vs. 25% PBS	**6.15E-04**	PLA vs. 25% PBS	**3.25E-02**		
PLA vs 5% PBS	**6.15E-04**	PLA vs 5% PBS	**3.25E-02**		
PLA vs. 5% PCL	**1.23E-03**	PLA vs. 5% PCL	1.30E-01		
PLA vs. 5% PTMC	7.55E-01	PLA vs. 5% PTMC	1.00		
PBS vs. 25% PBS	**6.15E-04**	PBS vs. 25% PBS	1.00		
PBS vs. 5% PBS	**2.48E-03**	PBS vs. 5% PBS	1.00		
PBS vs. 5% PCL	**6.15E-04**	PBS vs. 5% PCL	1.30E-01		
PBS vs. 5% PTMC	**6.15E-04**	PBS vs. 5% PTMC	**3.25E-02**		
25% PBS vs. 5% PBS	1.00	25% PBS vs. 5% PBS	1.00		
25% PBS vs. 5% PCL	**4.13E-02**	25% PBS vs. 5% PCL	1.30E-01		
25% PBS vs. 5% PTMC	**6.15E-04**	25% PBS vs. 5% PTMC	**3.25E-02**		
5% PBS vs. 5% PCL	7.55E-01	5% PBS vs. 5% PCL	3.90E-01		
5% PBS vs. 5% PTMC	**1.85E-02**	5% PBS vs. 5% PTMC	**3.25E-02**		
5% PCL vs. 5% PTMC	1.59E-01	5% PCL vs. 5% PTMC	1.00		

Supplementary Table S2. The exact Bonferroni corrected p-values obtained with the non-parametric Mann-Whitney test for material mechanical testing data. The following Bonferroni coefficients (the number of comparisons) were used: 5 for comparisons within a time point ad 6 for comparisons between time points. p-values<0.05 are presented in bold.

**Young's modulus: 0 week**		**Young's modulus: 1 week**		**Young's modulus: 2 week**	
comparison	***p-value***	***comparison***	***p-value***	***comparison***	***p-value***
PLA vs. PBS	1.79E-01	PLA vs. PBS	**3.97E-02**	PLA vs. PBS	**3.97E-02**
PLA vs. 25% PBS	3.57E-01	PLA vs. 25% PBS	**3.97E-02**	PLA vs. 25% PBS	1.00
PLA vs 5% PBS	1.00	PLA vs 5% PBS	1.00	PLA vs 5% PBS	1.00
PLA vs. 5% PCL	1.00	PLA vs. 5% PCL	1.00	PLA vs. 5% PCL	1.00
PLA vs. 5% PTMC	1.79E-01	PLA vs. 5% PTMC	**3.97E-02**	PLA vs. 5% PTMC	**3.97E-02**
**Young's modulus: 3 week**		**Young's modulus: 4 week**		**Young's modulus: 0 vs. 1 week**	
comparison	***p-value***	***comparison***	***p-value***	***comparison***	***p-value***
PLA vs. PBS	**3.97E-02**	PLA vs. PBS	7.94E-02	PLA	1.00
PLA vs. 25% PBS	**3.97E-02**	PLA vs. 25% PBS	7.54E-01	PBS	1.00
PLA vs 5% PBS	1.00	PLA vs 5% PBS	1.00	25% PBS	**4.76E-02**
PLA vs. 5% PCL	1.00	PLA vs. 5% PCL	1.00	5% PBS	1.00
PLA vs. 5% PTMC	**3.97E-02**	PLA vs. 5% PTMC	**3.97E-02**	5% PCL	1.00
				5% PTMC	1.00
**Young's modulus: 0 vs. 2 week**		**Young's modulus: 0 vs. 3 week**		**Young's modulus: 0 vs. 4 week**	
comparison	***p-value***	***comparison***	***p-value***	***comparison***	***p-value***
PLA	1.00	PLA	1.00	PLA	1.00
PBS	1.00	PBS	1.00	PBS	1.00
25% PBS	1.90E-01	25% PBS	1.90E-01	25% PBS	9.52E-02
5% PBS	5.71E-01	5% PBS	1.00	5% PBS	1.00
5% PCL	9.05E-01	5% PCL	1.00	5% PCL	1.00
5% PTMC	1.00	5% PTMC	1.00	5% PTMC	1.00
**Strain at break: 0 week**	**Young's modulus: 1 week**	**Strain at break: 2 week**	**Strain at break: 0 week**	**Strain at break: 1 week**	**Strain at break: 2 week**
comparison	***p-value***	***comparison***	***p-value***	***comparison***	***p-value***
PLA vs. PBS	**3.97E-02**	PLA vs. PBS	**03.97E-02**	PLA vs. PBS	**3.97E-02**
PLA vs. 25% PBS	1.59E-01	PLA vs. 25% PBS	1.00	PLA vs. 25% PBS	1.59E-01
PLA vs 5% PBS	7.94E-02	PLA vs 5% PBS	1.00	PLA vs 5% PBS	4.76E-01
PLA vs. 5% PCL	4.76E-01	PLA vs. 5% PCL	1.00	PLA vs. 5% PCL	1.59E-01
PLA vs. 5% PTMC	7.94E-02	PLA vs. 5% PTMC	**3.97E-02**	PLA vs. 5% PTMC	**3.97E-02**
**Strain at break: 3 week**	**Strain at break: 4 week**	**Strain at break: 0 vs. 1 week**	**Strain at break: 3 week**	**Strain at break: 4 week**	**Strain at break: 0 vs. 1 week**
comparison	***p-value***	***comparison***	***p-value***	***comparison***	***p-value***
PLA vs. PBS	**3.97E-02**	PLA vs. PBS	**3.97E-02**	PLA	1.00
PLA vs. 25% PBS	1.00	PLA vs. 25% PBS	2.78E-01	PBS	1.00
PLA vs 5% PBS	1.00	PLA vs 5% PBS	**3.97E-02**	25% PBS	1.00
PLA vs. 5% PCL	**3.97E-02**	PLA vs. 5% PCL	**3.97E-02**	5% PBS	1.00
PLA vs. 5% PTMC	7.94E-02	PLA vs. 5% PTMC	1.00	5% PCL	9.05E-01
				5% PTMC	1.00
**Strain at break: 0 vs. 2 week**	**Strain at break: 0 vs. 3 week**	**Strain at break: 0 vs. 4 week**	**Strain at break: 0 vs. 2 week**	**Strain at break: 0 vs. 3 week**	**Strain at break: 0 vs. 4 week**
comparison	***p-value***	***comparison***	***p-value***	***comparison***	***p-value***
PLA	9.05E-01	PLA	1.00	PLA	9.05E-01
PBS	1.00	PBS	3.33E-01	PBS	1.00
25% PBS	1.00	25% PBS	1.00	25% PBS	1.00
5% PBS	5.71E-01	5% PBS	1.00	5% PBS	3.33E-01
5% PCL	1.00	5% PCL	1.00	5% PCL	1.00
5% PTMC	1.00	5% PTMC	1.00	5% PTMC	1.00
